# Protective effects of synbiotic diets of *Bacillus coagulans*, *Lactobacillus plantarum* and inulin against acute cadmium toxicity in rats

**DOI:** 10.1186/s12906-017-1803-3

**Published:** 2017-06-05

**Authors:** Dornoush Jafarpour, Seyed Shahram Shekarforoush, Hamid Reza Ghaisari, Saeed Nazifi, Javad Sajedianfard, Mohammad Hadi Eskandari

**Affiliations:** 1 Department of Food Hygiene and Public Health, School of Veterinary Medicine, Shiraz University, Shiraz, Iran; 2 Department of Clinical Pathology, School of Veterinary Medicine, Shiraz University, Shiraz, Iran; 3 Department of Physiology, School of Veterinary Medicine, Shiraz University, Shiraz, Iran; 4 Department of Food Science and Technology, College of Agriculture, Shiraz University, Shiraz, Iran

**Keywords:** Cadmium, *Lactobacillus plantarum*, *Bacillus Coagulans*, Inulin, Oxidative stress

## Abstract

**Background:**

Cadmium is a heavy metal that causes oxidative stress and has toxic effects in humans. The aim of this study was to investigate the influence of two probiotics along with a prebiotic in preventing the toxic effects of cadmium in rats.

**Methods:**

Twenty-four male Wistar rats were randomly divided into four groups namely control, cadmium only, cadmium along with Lactobacillus plantarum (1 × 109 CFU/day) and inulin (5% of feedstuff) and cadmium along with Bacillus coagulans (1 × 109 spore/day) and inulin (5% of feedstuff). Cadmium treated groups received 200 μg/rat/day CdCl2 administered by gavage. During the 42-day experimental period, they were weighed weekly. For evaluation of changes in oxidative stress, the levels of some biochemicals and enzymes of serum including SOD, GPX, MDA, AST, ALT, total bilirubin, BUN and creatinine, and also SOD level of livers were measured at day 21 and 42 of treatment. The cadmium content of kidney and liver was determined by using atomic absorption mass spectrophotometry. Data were analyzed using analysis of variance (ANOVA) followed by Duncan’s post hoc test.

**Results:**

Treatment of cadmium induced rats with synbiotic diets significantly improved the liver enzymes and biochemical parameters that decreased AST, ALT, total bilirubin, BUN and metal accumulation in the liver and kidney and increased body weight, serum and liver SOD values in comparison with the cadmium-treated group. No significant differences were observed with MDA and GP_X_ values between all groups (*p* > 0.05).

**Conclusions:**

This study showed that synbiotic diets containing probiotics (*L. plantarum* and *B. coagulans)* in combination with the prebiotic (inulin) can reduce the level of cadmium in the liver and kidney, preventing their damage and recover antioxidant enzymes in acute cadmium poisoning in rat.

**Electronic supplementary material:**

The online version of this article (doi:10.1186/s12906-017-1803-3) contains supplementary material, which is available to authorized users.

## Background

Cadmium is a trace element and is one of the non-essential metals that has toxic effects in man [1]. It is a major concern for public health and is present at low concentration in soil, rock and drinking water. Cadmium can mainly be found in the earth’s crust. It always occurs in combination with zinc. Cadmium also consists in the industries as an inevitable by-product of zinc, lead and coppe extraction. Because of its highly soluble nature compared to other metals, cadmium is taken up by plants and is stored in food and feed products and human receives it mainly through food [2]. Foodstuffs such as mushrooms, shellfish, mussels, liver, cocoa powder and dried seaweed that are rich in cadmium. They can greatly increase the cadmium concentration in the human body. Cigarette smoke transports high levels of cadmium into the lungs. Blood then transports the cadmium through the rest of the body where it can increase its toxic effects by potentiating with the cadmium that is already present from cadmium-rich food.

Cadmium is first transported to the liver through the blood stream where, it binds to proteins to form complexes that are transported to the kidneys. Cadmium accumulates in the kidneys where it damages the kidney’s filtering mechanisms. This causes the excretion of essential proteins and sugars from the body and further kidney damage. The World Health Organization (WHO) specifies the tolerable intake for cadmium at 7 μg/kg of bodyweight/week. Dietary exposure to large cadmium doses has been reported to result in adverse health effects in the kidneys, liver, bone, mammary gland, breast, pancreas, colon [3–5] and also neurological alterations in humans such as lower attention, hypernociception, olfactory dysfunction and memory deficits [6].

Numerous studies have revealed that the mentioned metal caused oxidative stress by inducing the generation of reactive oxygen species (ROS). It has been assumed that cadmium reduced antioxidant defence system of cells via glutathione depletion, affecting some enzymes such as glutathione peroxidase (GP_X_), superoxide dismutase (SOD) and increasing lipid, protein and DNA oxidation [7, 8].

Chelation therapy has been applied for heavy metals poisoning excretion in therapeutic strategy. Evidence has shown that these chemical chelators (like CaNa_2_ EDTA) have some side effects such as nausea, vomiting, anorexia, appetite loss, and diarrhoea [9]. Therefore, in recent years, there has been an increasing number of studies to find safe and efficient dietary compounds against heavy metals toxicity.

Synbiotic refers to a nutritional supplement which combines probiotic and prebiotic in a form of synergism [10]. Prebiotics are typically non-digestible fibre compounds that pass undigested through the gastrointestinal tract and stimulate the growth and activity of advantageous bacteria like probiotics [10]. Probiotics are live bacteria which are intended to colonize the large intestine, avoid the adherence of pathogens and confer physiological health benefits to the host [11]. Lactic acid bacteria (LAB) such as *Lactobacillus acidophilus*, *L. plantarum* and *Bifidobacterium* are known to be the most common probiotics. These probiotics are very sensitive to normal physiological conditions such as the very low pH of the stomach and bile salts [12]. Hence, a novel beneficial probiotic is introduced that can survive under extreme conditions. Some strains of *Bacillus coagulans* are able to withstand the gastrointestinal tract and continue their metabolic activities via spore production [13, 14].

Probiotic bacteria may have the tendency for toxin protection due to their abilities in heavy metals binding and antioxidant effect. It is reported that *L. rhamnosus* LC105 and *L. plantarum* ID9263 can bind and remove heavy metals such as lead, cadmium and copper from polluted water in vitro [15, 16]. Also, the positive effect of *L. plantarum* CCFM8661 on reducing the toxicity of lead in mice was demonstrated by Tian et al. [17]. Al-Wabel et al. [18] used synbiotic fermented milk against lead acetate contamination in rats and found out the effective role of this diet on the liver by increasing the activity of the antioxidant enzymes.

The purpose of the present study is to compare the effect of two probiotic, *L. plantarum* and *B. coagulans*, in combination with the prebiotics, inulin, to prevent the toxic effects of cadmium in rats.

## Methods

### Preparing suspension of probiotic bacteria

Two probiotic bacteria used throughout the study were *B. coagulans* and *L. plantarum*. Lyophilized probiotic *B. coagulans* was generously supplied by the Pardis Roshd Mehregan Company, Shiraz, Iran. *B. coagulans* spores were prepared according to the method of Abhari et al. [19]. The bacteria were grown aerobically in nutrient yeast extract salt medium (NYSM) agar. After incubation at 37 °C for 24 h, a single colony was obtained, inoculated into NYSM broth and kept in a shaker incubator at 37 °C for 48 h. Bacterial cells was achieved by centrifugation at 3000×g for 20 min and then heated at 80 °C for 15 min to kill the vegetative cells. Finally, spore suspension of the bacterium was prepared at a concentration of 1 × 10^9^ spore/ml in sterile saline and kept in the refrigerator up to a week [19].

*L. plantarum* CNR273 was taken from the culture collection of the Department of Food Science and Technology, Shiraz University, Iran. It was cultivated aerobically in De Man Rogosa Sharpe (MRS) agar (Difco, Detroit, MI, USA) at 37 °C for 48 h. A single colony was inoculated into MRS broth and incubated at 37 °C with shaking for 48 h. The bacterial pellets were attained by centrifugation at 3000×g for 20 min. The viable bacterial cells were prepared at a concentration of 1 × 10^9^ CFU/mL after appropriate serial dilution and plating in MRS agar. This method was carried out as described by Jafarpour et al. [20]. The bacterial suspension was kept in the refrigerator up to a week.

### Preparation of cadmium

Cd-exposed groups received cadmium chloride (CdCl_2_) (Merck, Darmstadt, Germany) solution (200 μg/mL) at a dose of 200 μg/rat/day. The cadmium solution at the mentioned dose was prepared by the method described by Nwokocha et al. [21]. The molecular weight of CdCl_2_ was divided by the molecular weight of cadmium (183.32/112.41) to obtain 1.63 g as the weight of 1 part of cadmium in CdCl_2_. 0.326 g of cadmium chloride was dissolved in 1 L of distilled water to achieve a concentration of 200 ppm. The CdCl_2_ solution was fed daily to each rat by gavage.

### Animals and treatment

Twenty-four male Wistar rats weighing approximately 170 ± 10 g were used. Animals were purchased from the Razi Vaccine and Serum Research Institute, Shiraz, Iran and kept in standard polypropylene cages under the following conditions: temperature (23 ± 2 °C), relative humidity (38%), and exposure to a 12 h light/dark cycle with ad libitum access to food and tap water.

After an acclimatization period of 1 week, the animals were randomly divided into four groups (*n* = 6/group). The rats were identified by marking their tails. Three rats of each group were treated for 21 days and the treatment of the other three were continued for 42 days. They were weighed individually every week by using a balance scale (Mettler Toledo® scale, model Spider 2). Table 1 summarizes the profile of rat groups and fed diets.

**Table 1:** Treatment groups used in the experimental study

Treatment groups	Feeding	Gavaging (1 mL volume, once daily)
Control	Standard diet	Normal saline
Cd	Standard diet	Cadmium (200 μg/mL)
ILp + Cd	Standard diet + 5% inulin	*L. plantarum* (1 × 10^9^ CFU/mL) + cadmium
IBc + Cd	Standard diet + 5% inulin	*B. coagulans* (1 × 10^9^ spore/mL) + cadmium

The standard pellet feedstuff contained 17.9% protein, 48.5% starch, 4.9% sugar, 5.3% crude fiber, 4.6% fat and 7.1% ash. 5% chicory based inulin (Roosendaal, The Netherlands) was added to the diet of the two treatment groups.

### Collection of blood and organ samples

On days 21 and 42, three rats from each treatment group were sacrificed under an atmosphere of 100% diethyl ether anaesthesia after overnight fasting. Two blood samples were immediately obtained via the right side of the heart for biochemical assay. One sample was transferred to an EDTA tube and the second one was transferred to a plain tube without anticoagulant and allowed to stand for 2 h. Thereafter the samples were centrifuged at 2000×g for 10 min to harvest plasma and serum, respectively. A small amount of the samples were also heparinized and used for preparation of red blood cell hemolysate for SOD and GPx assay.

Livers and kidneys of sacrificed rats were removed from the bodies, washed in ice-cold saline, and stored at −80 °C for further measurements.

### Superoxide dismutase (SOD) assay

Liver samples (0.5 g) were weighed and homogenized on ice and in cold 0.5 M phosphate buffer (pH 7.2) by using a homogenizer (Yellow Line DI18, Ikawerke, Germany). The homogenates were then centrifuged at 3000×*g* for 15 min at 4 °C (Hettichmikro 200R, Tuttlingen, Germany) and the supernatants were stored at −80 °C until use.

Blood and liver SOD activity was evaluated with SOD detection RANSOD kit (Randox Lab., Crumlin United Kingdom) according to the manufacturer’s instructions. SOD levels were recorded at 505 nm through a standard curve and expressed as unit per gram of hemoglobin or liver tissue.

### Glutathione peroxidase (GP_X_) assay

The activity of GP_X_ was evaluated with GPx detection RANSEL kit (Randox Lab., Crumlin United Kingdom) according to the manufacturer’s instructions. GP_X_ levels were measured spectrophotometrically at 340 nm and expressed as unit per gram of hemoglobin (U/g Hb). One unit (U) of GP_X_ activity was defined as the amount of enzyme that converts 1 μmol of NADPH to NADP^+^ per minute.

### Measurement of lipid peroxidation (MDA)

To evaluate lipid peroxidation in serum, a modified HPLC method was used based on the reaction of malondialdehyde (MDA) with thiobarbituric acid (TBA) to form a colored MDA–TBA adduct [22]. Forty μL of the sample was diluted with 100 μL of H_2_O and mixed with 20 μL of 2.8 mmol/L butylated hydroxyl toluene (BHT) in ethanol, 40 μL of 81 g/L sodium dodecyl sulfate and 600 μL of TBA reagent (8 g/L TBA diluted 1:1 with 200 ml/L acetic acid adjusted to pH 3.5 with NaOH). The mixture was immediately heated (60 min at 95 °C) and cooled with running water; 200 μL of H_2_O and 1000 μL of butanol–pyridine (15:1, *v*/v) were added afterwards. After vigorous mixing, the organic layer was separated by centrifugation (3 min at 16,000×g). The supernatant was analyzed on a UV-visible spectrophotometer fitted with an 80 μL flow cell. The absorbance was measured at 532 nm (the mobile phase consisted of 300 mL/L methanol in 50 mmol/L potassium dihydrogen phosphate buffer, pH 7.0). 1, 1, 3, 3-tetraethoxypropane was used as a standard, and MDA-TBA reactive substances’ values were expressed as MDA mmol/L. The HPLC system consisted of a solvent delivery pump (JASCO 980-PU, Tokyo, Japan), a reversed-phase column (Luna C18, 250 mm × 4.6 mm, Phenomenex, CA, USA), and a UV–Vis detector (Jasco, UV-975, Tokyo, Japan) operated at 532 nm.

### Serum biochemical analyses

All biochemical parameters including alanine aminotransferase (ALT), aspartate aminotransferase (AST), creatinine, blood urea nitrogen (BUN) and bilirubin were measured by commercial kits (Pars Azmoon Co., Tehran, Iran). All the enzyme activities were measured at 37 °C and the results have been presented in units per litre [23]. Biochemical analyses were measured using a standard autoanalyser with veterinary software (Cobas-Mira, ABX-Diagnostics, Japan).

### Cadmium measurement

Approximately 0.5 g of liver and kidney samples were taken from the experimental rats at day 42, weighed with an accuracy of 0.01 g and homogenized using a blade mixer until the appropriate consistency was reached. Then the homogenized tissues were digested in 10 mL of concentrated nitric acid at boiling bath (about 96 °C) for 18 h [24]. The concentration of cadmium was then determined by atomic absorption mass spectrophotometry (Shimadzu-AA-670, Japan).

### Animal ethics

All the procedures conducted in this experiment follow the ethical guidelines of animal welfare approved by the Ethics Committee of the School of Veterinary Medicine, Shiraz University, Shiraz, Iran (Ethical approved number: 1392/905659).

### Statistical analysis

The results are expressed as mean ± SD with a significance level of *p* < 0.05. Statistical analysis for significant differences among group means were tested by analysis of variance, followed by Duncan’s post hoc test with the help of SPSS 16.0 Windows software.

## Results

### Changes in body weight

**Fig. 1:**
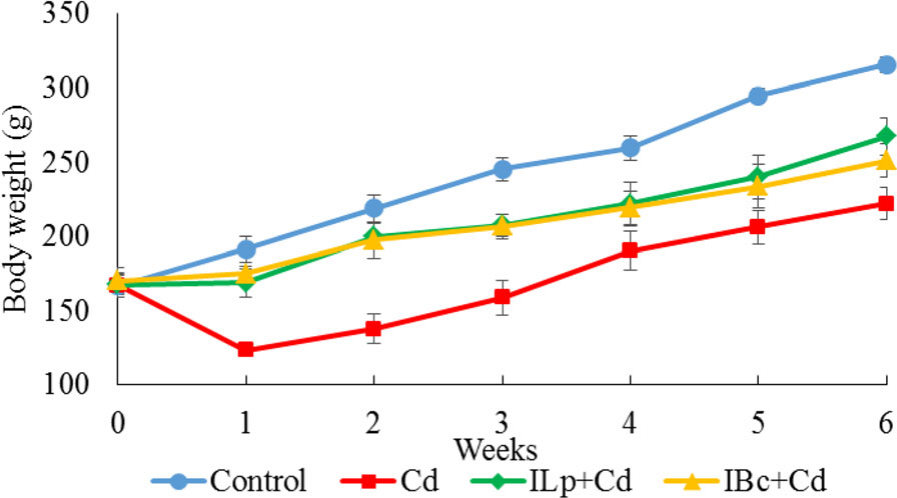
Effects of oral administration of cadmium on body weight of rat fed synbiotic diets containing inulin, *B. coagulans* and *L. plantarum* following 6 weeks of treatment. Cd: cadmium; ILp + Cd: inulin, *Lactobacillus plantarum* and cadmium; IBc + Cd: inulin, *Bacillus coagulans* and cadmium. All results are expressed as mean and standard errors of 6 rats in each group

Figure 1 shows the changes to the body weight of the different groups during the experimental time. There was a gradual increase in the body weight of all groups over 6 weeks but a significant reduction was observed in the body weight of the cadmium group at the start of the experiment (*p* < 0.05). After a period of three weeks, the cadmium treated animals exhibited sluggish behaviour and had the lowest weight amongst the other three treatment groups. Data indicates that the treatment of the animals with synbiotics (198.25 ± 8.88 and 207.75 ± 6.34 g, respectively, for ILp + Cd and IBc + Cd groups) significantly (*p* < 0.05) increased their body weights compared with cadmium group (168.75 ± 8.58 g).

### Effects on biochemical properties

The effects of probiotics and inulin as a prebiotic on oxidative stress indicators (SOD, GP_X_ and MDA) are shown in Table 2. At day 42 of the experiment, cadmium treated rats showed a significant decrease in the activity of the enzyme SOD both in the blood and liver (254.2 ± 1.60 and 2.55 ± 0.04 U/g, respectively), compared to other groups. Rats treated with Cd + synbiotic diet showed a significant increase in the activity of blood SOD (290.50 ± 3.20 and 287.00 ± 4.2 U/g Hb, respectively, for ILp + Cd and IBc + Cd groups) and liver SOD (2.74 ± 0.02 and 2.69 ± 0.03 U/g liver tissue, respectively, for ILp + Cd and IBc + Cd groups) when compared to cadmium treated rats (*p* < 0.05).

**Table 2:** Effects of oral administration of cadmium on levels of glutathione peroxidase (GPx), superoxide dismutase (SOD), liver superoxide dismutase (liver SOD) and malondialdehyde (MDA) of rat fed synbiotic diets containing inulin, *B. coagulans* and *L. plantarum* at days 21 and 42 of treatments

Treatments	GP_X_ (U/g Hb)		Blood SOD (U/g Hb)		Liver SOD (U/g tissue)		MDA (mmol/L)	
	Day21	Day42	Day21	Day42	Day21	Day42	Day21	Day42
Control	1178 ± 168^aA^	1122 ± 257^aA^	300.9 ± 2.8^aA^	301.8 ± 1.6^aA^	2.73 ± 0.03^aA^	2.89 ± 0.04^aB^	4.96 ± 0.09^aA^	4.99 ± 0.03^aA^
Cd	1402 ± 97^aA^	1122 ± 194^aA^	250.0 ± 1.6^bA^	254.2 ± 1.6^bA^	2.53 ± 0.03^bA^	2.55 ± 0.04^bA^	4.98 ± 0.06^aA^	5.00 ± 0.03^aA^
ILp + Cd	1234 ± 194^aA^	1009 ± 168^aA^	290.4 ± 4.8^cA^	290.5 ± 3.2^acA^	2.70 ± 0.03^cA^	2.74 ± 0.02^cB^	4.99 ± 0.05^aA^	4.97 ± 0.07^aA^
IBc + Cd	1290 ± 97^aA^	1066 ± 350^aA^	286.1 ± 2.7^cA^	287.0 ± 4.2^cA^	2.67 ± 0.06^cA^	2.69 ± 0.03^cA^	5.00 ± 0.02^aA^	4.98 ± 0.05^aA^

*Cd* cadmium, *ILp + Cd* inulin, *Lactobacillus plantarum* and cadmium, *IBc + Cd*, inulin, *Bacillus coagulans* and cadmium. Values are expressed as mean ± SD. The different small letters indicate statistically significant differences in columns (*P* < 0.05). The different capital letters indicate statistically significant differences between days in each parameter (*P* < 0.05).

No significant differences were observed with regard to MDA contents in the treatment groups during the 42 days. Similarly, GP_X_ exhibited no differences between groups and also during the different times.

**Fig. 2:**
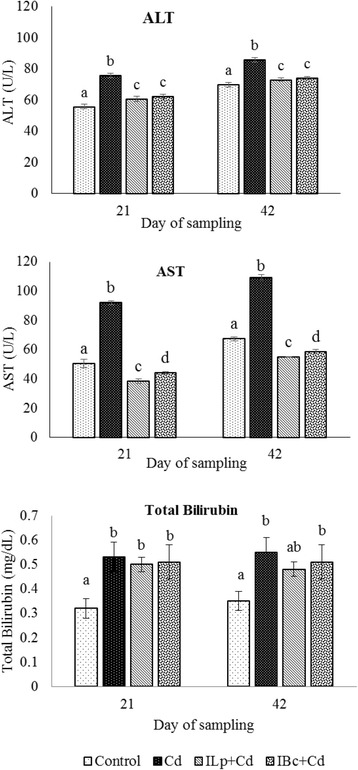
Effects of oral administration of cadmium on levels of ALT, AST and total bilirubin of rats fed symbiotic diets at days 21 and 42 of treatments. Cd: cadmium; ILp + Cd: inulin, *Lactobacillus plantarum* and cadmium; IBc + Cd: inulin, *Bacillus coagulans* and cadmium. All results are expressed as mean and standard deviation. The different letters indicate statistically significant differences (*P* < 0.05)

Serum activities of ALT, AST and total bilirubin were significantly higher in the cadmium treated group when compared to other groups (*p* < 0.05) (Fig. 2). Also, the data showed that probiotics along with inulin had significantly lower ALT and AST values in comparison with the cadmium treated group (*p* < 0.05) but that they had no effect on total bilirubin compared to the Cd group (Fig. 2).

Creatinine and BUN levels in the control group at day 21 and 42 of treatment were 0.72 and 0.80, 18.38 and 19.91 mg/dL, respectively (Table 3). Both factors were significantly higher in cadmium induced rat (*p* < 0.05). Administration of *L. plantarum* and *B. coagulans* together with inulin in cadmium induced rat caused a marked decrease in the level of creatinine, at day 42 of experiment, from 1.11 to 0.91 and 0.99 mg/dL, respectively (*p* < 0.05) (Table 3). The level of BUN also significantly decreased in these groups from 24.55 to 20.29 and 20.67 mg/dL respectively (*p* < 0.05) (Table 3).

**Table 3:** Effects of oral administration of cadmium on levels of BUN and creatinine of rats fed synbiotic diets containing inulin, *B. coagulans* and *L. plantarum* at days 21 and 42 of treatments

Treatments	Creatinine (mg/dL)		BUN (mg/dL)	
	Day21	Day42	Day21	Day42
Control	0.72 ± 0.10^aA^	0.80 ± 0.03^aA^	18.38 ± 0.73^aA^	19.91 ± 0.70^aA^
Cd	1.20 ± 0.17^bA^	1.11 ± 0.20^bA^	23.59 ± 0.64^bA^	24.55 ± 0.50^bA^
ILp + Cd	0.95 ± 0.12^cA^	0.91 ± 0.05^acA^	19.44 ± 1.03^aA^	20.29 ± 0.42^aA^
IBc + Cd	0.99 ± 0.14^cA^	0.99 ± 0.05^bcA^	19.89 ± 0.34^aA^	20.67 ± 0.36^aA^

*Cd* cadmium, *ILp + Cd* inulin, *Lactobacillus plantarum* and cadmium, *IBc + Cd* inulin, *Bacillus coagulans* and cadmium. Values are expressed as mean ± SD. The different small letters indicate statistically significant differences in columns (*P* < 0.05). The different capital letters indicate statistically significant differences between days in each parameter (*P* < 0.05).

### Effects of synbiotic diet on heavy metals contents in the liver and kidney

**Fig. 3:**
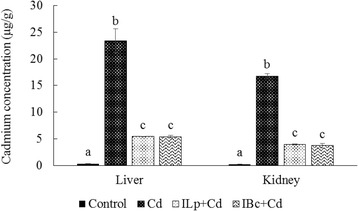
Effect of synbiotic diet on the accumulation of cadmium in liver and kidney of rats at day 42 of treatments. Cd: cadmium; ILp + Cd: inulin, *Lactobacillus plantarum* and cadmium; IBc + Cd: inulin, *Bacillus coagulans* and cadmium. All results are expressed as mean and standard deviation. The different letters indicate statistically significant differences (*P* < 0.05)

The results of the cadmium content of both the liver and kidney of the treated groups are presented in Fig. 3. When a synbiotic diet was administered together with the cadmium, it provided to significantly lower metal accumulation compared to the cadmium group (*p* < 0.05). In ILp + Cd and IBc + Cd groups, the cadmium level of the livers were significantly decreased from 23.36 ± 2.27 to 5.44 ± 0.04 and 5.43 ± 0.27 μg/g, respectively (*p* < 0.05). A similar trend was also seen in the cadmium level of the kidney (Fig. 3).

## Discussion

Heavy metal poisoning has become a major health concern in industrialized countries. Cadmium is one of the most toxic heavy metal and is so harmful that strategies must be devised to reduce its levels and hence toxicity. Various studies have reported that specific microorganisms such as LAB are able to absorb several metal ions and help to eliminate them [25].

In the present study, two probiotic bacteria (*B. coagulans* and *L. plantarum*) in combination with inulin were used to reduce the toxic effects of cadmium. Herein, we observed that two symbiotic diets could offer significant protection against cadmium toxicity in vivo by decreasing cadmium in the liver and kidney, and thus preventing alterations in the levels of SOD, ALT, AST, BUN and creatinine. There are reports presented that indicate *B. longum* 46 and *L. fermentum* ME3 can bind the toxic cathionic heavy metals, cadmium and lead from water [15]. Tian et al. [17] noted that *L. plantarum* CCFM8661 had the potency to alleviate the lead toxicity in mice. In addition to cadmium reduction, the protective effects on the antioxidant enzymes and oxidative stress were studied.

It was proposed that animals experiencing continuous exposure to heavy metals usually lose weight [21]. In this study we observed that cadmium administration resulted in remarkable reduction in body weight of rats but at first three weeks of experiment weight losses were significantly reversed by synbiotic treatment. Similar types of findings were observed by Horton et al. [26] and Tatara et al. [27].

The SOD is an important antioxidant defence mechanism that plays an extremely important role in the protection of all aerobic life-systems against oxygen toxicity. The main function of this enzyme is to accelerate the dismutation of the toxic superoxide produced during oxidative energy processes to hydrogen peroxide and molecular oxygen and reduces the oxidative stress. We realized that the synbiotic diets resulted in increasing the SOD enzyme against cadmium toxicity. AL-Hashem [28] reported significant reduction in SOD activity of rats that were exposed to aluminium chloride. Similar assumption was proposed by Tian et al. [17].

In the present study, despite the accumulation of cadmium in the liver (23.36 ± 2.27 μg/g) and kidney (16.77 ± 0.48 μg/g) of the rats which were taken 200 mg/day of CdCl_2_ for 42 days, concentrations of the GPx and MD as major markers of oxidative stress were not raised compared to the control group.

Serum ALT and AST are the most commonly used biochemical markers of liver damage and are considered sensitive indicators of hepatic injury. The marked treatment increased the activities of serum ALT and AST induced liver injury due to metals exposure [29]. We perceived that the activities of serum ALT and AST increased due to cadmium toxicity. In contrast, in synbiotic groups, liver injury improved as a result of decreased serum ALT and AST levels. The report of Al-Wabel et al. [18] that utilization of synbiotic fermented milk containing *Lactobacillus acidophilus* and *Bifidobacterium bifidum* decreased serum ALT and AST levels in rats exposed to lead acetate is in agreement with our findings.

From the results, both AST and ALT values decreased in synbiotic treatment groups but AST was even lower than the control group. The baseline of AST activity is usually in a defined range due to hepatic and muscular cells death in control group [30]. It seems, the death trend of hepatic and muscular cells and the AST level were significantly reduced in the rats treated with synbiotics. This finding suggests the protective effect of synbiotic diet.

To detect the renal damage the creatinine and BUN concentrations in serum were examined, as they are recognized as biomarkers of renal injury and the amplification of these biomarkers is usually correlated with impairment of renal function [31, 32]. In the Cd group the values of creatinine and BUN increased but in the ILp + Cd and IBc + Cd groups their values decreased. Therefore our study showed that synbiotic treatment decreased elevated creatine and BUN level induced by cadmium exposure. Ulutas et al. [33] reported renal cell injury by cadmium, Cr resulted in elevation of serum urea and creatinine in metal treated rats compared to the negative control.

Our results showed that, although under continuous exposure to cadmium, the concentration of this heavy metal increased in the kidney and liver and that a synbiotic diet played a crucial role in the reduction of metal accumulation. Also, comparison between two probiotics showed both *L. plantarum* and *B. coagulans* had a major effect against cadmium toxicity and no significant differences were observed between them. Previous research has indicated that diet may influence heavy metal uptake and/or excretion [34]. Gram - positive bacteria, particularly Bacillus and LAB spp., have high adsorptive capacity of heavy metals due to high peptidoglycan and teichoic acid content in their cell walls [35, 36]. Surface anionic groups in gram-positive bacteria are effective in the removal of heavy metals. Reduction of cadmium accumulation in the liver and kidney and increased levels in the stool of challenged groups indicated the ability of the probiotic bacteria to bind with cadmium and reduce the absorption of this heavy metal through the intestine by defecation. These observations are also in agreement with earlier studies by Tian et al. [17], Majlesi et al. [37] and Zhai et al. [38].

## Conclusion

This study showed that synbiotic diets which is the combination of probiotics (*L. plantarum* and *B. coagulans)* and prebiotic (inulin) can reduce the level of cadmium in the tissues, preventing liver and kidney damage and recover antioxidant enzymes in acute cadmium poisoning in rat. Our results suggest that the two synbiotic diet may be therapeutic in the treatment and prevention of cadmium poisoning in the high risk regions.

## Additional file

Additional file 1: Raw data. (XLS 452 KB)

## Abbreviations

ALT: alanine aminotransferase
AST: aspartate aminotransferase
Cd: cadmium
GP_X_: Glutathione peroxidase
IBc + Cd: inulin, *Bacillus coagulans* and cadmium
ILp + Cd: inulin, *Lactobacillus plantarum* and cadmium
MDA: malondialdehyde
SOD: Superoxide dismutase
